# Association of preoperative handgrip strength and postoperative recovery with outcomes in cardiac surgery patients ≥60 years old

**DOI:** 10.1016/j.xjon.2026.101612

**Published:** 2026-02-09

**Authors:** Thadakorn Tantisarasart, Narisara Rachapongthai, Jutarat Tanasansuttiporn, Orarat Karnjanawanichkul, Suttasinee Petsakul, Laortip Rattanpittayaporna, Pongsanae Duangpakdee, Sirichai Cheewatanakornkul, Khantaros Saelim

**Affiliations:** a Department of Anesthesiology, Faculty of Medicine, Prince of Songkla University, Songkhla, Thailand; b Department of Surgery, Faculty of Medicine, Prince of Songkla University, Songkhla, Thailand; c Department of Internal Medicine, Faculty of Medicine, Prince of Songkla University, Songkhla, Thailand

**Keywords:** cardiac surgery, elderly, handgrip strength, postoperative complication

## Abstract

**Background:**

Cardiac surgery is a critical treatment option for patients with cardiovascular disease; however, it carries a significant risk of complications. The aim of this study was to investigate the association between changes in handgrip strength and postoperative complications in older adults undergoing cardiac surgery to establish handgrip strength as a practical tool for improving surgical outcomes.

**Methods:**

This prospective cohort study included 105 patients aged 60 years or more who underwent cardiac surgery. We evaluated whether handgrip recovery (handgrip strength on postoperative day 5/preoperative handgrip strength) was superior to absolute handgrip strength in predicting 30-day postoperative complications. Logistic regression analysis was performed to determine the association between handgrip recovery and the incidence of postoperative complications.

**Results:**

Among the 105 patients, 65 experienced 30-day postoperative complications. The area under the receiver operating characteristic curve for handgrip recovery as a predictor of complications was 0.765 (*P* < .001), with an optimal cutoff value of 86.67%. Patients with lower grip recovery showed a 10.56-fold higher risk of 30-day postoperative complications than those with normal recovery. When patients were compared using the cutoff value for handgrip recovery, those in the low recovery group exhibited significantly poorer outcomes, including a longer intensive care unit stay (4 vs 3 days, *P* = .007) and longer hospitalization (8 vs 7 days, *P* = .002). Logistic regression analysis demonstrated a statistically significant association between handgrip recovery and the incidence of postoperative complications.

**Conclusions:**

Low handgrip recovery is a superior predictor of postoperative complications, including longer intensive care unit and hospitalization durations, in older patients who undergo cardiac surgery under cardiopulmonary bypass.

**Figure:**
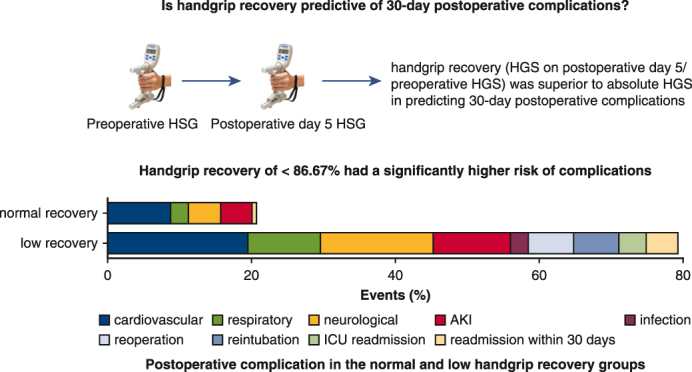
Handgrip recovery is more predictive of 30-day complications than absolute HGS values.

**Central Message**

Handgrip recovery is a better predictor than absolute HGS for postoperative complications in patients undergoing cardiac surgery with cardiopulmonary bypass.

**Perspective**

Assessment of hand grip strength perioperatively could predict mortality and perioperative complications after various surgeries. We aim to identify the best predictor tool for hand grip strength in patients undergoing cardiac surgery with cardiopulmonary bypass to enhance recovery and reduce postoperative complications.

Cardiovascular disease is a leading cause of death worldwide and a significant public health concern. Cardiac surgery is a primary treatment modality for various cardiac conditions; however, it carries a substantial risk of postoperative complications, particularly those affecting the cardiovascular and respiratory systems. Older individuals are more likely to be frail and, consequently, to experience higher rates of morbidity and mortality. Recently, the postoperative outcome at 30 days has been widely recognized as an important indicator of recovery in the early stages, particularly after cardiac surgery. Increased levels of proinflammatory cytokines such as tumor necrosis factor-alpha and interleukin-6 after cardiac surgery contribute to muscle proteolysis, leading to postoperative muscle weakness.^1^^,^^2^

Handgrip strength (HGS) is a simple, cost-effective bedside measurement used to assess muscle strength. Evidence supports its value as a significant predictor of all-cause mortality within 12 months and prolonged intensive care unit (ICU) stay.^3^ Furthermore, a systematic review demonstrated that HGS is strongly associated with lung function in healthy adults.^4^ As an objective and practical measurement, HGS can be used to identify patients at a higher risk of mortality and delayed recovery after cardiac surgery.^5^ Recent observational studies have further reported that handgrip recovery is a stronger predictor of 30-day postoperative complications than either HGS or HGS normalized by body weight in cardiac surgery, indicating heterogeneity.^6^ There are no data on the absolute change in HGS or HGS recovery in older patients undergoing cardiac surgery with cardiopulmonary bypass. Therefore, the aim of this study was to investigate the association between changes in HGS and postoperative complications in older adults within this group.

## Material and Methods

This study was approved by the Office of Human Research Ethics Committee, Faculty of Medicine, Prince of Songkla University (REC 66-036-8-1, approved date June 12, 2023) and includes consent for publication. It was conducted at Songklanagarind Hospital, Thailand, from June 2023 to March 2024. A total of 105 consecutive patients aged 60 years or more undergoing cardiac surgery under cardiopulmonary bypass were enrolled during the study period. Patients with clinical instability, with upper-limb injuries or deformities, with upper-arm weakness, who underwent emergency surgery, and who refused to participate in the follow-up were excluded.

Informed consent was obtained from all participants. All patients did not receive any benzodiazepine or sedative agents. Anesthesia was maintained with inhalation agents in all patients. HGS was measured using a JAMAR Hydraulic Hand Dynamometer (Model J00105, Lafayette Instrument Company). It is regularly calibrated before using. The patients were instructed to sit in a comfortable position with their elbows extended to the side and squeeze the dynamometer with maximum strength. The highest value among 3 measurements for each hand was recorded and used for analysis. HGS was measured and recorded by an independent observer who did not participate in the study. Absolute HGS was defined as the preoperative HGS and HGS on postoperative day (POD) 5. Handgrip recovery was determined as the ratio of HGS on POD 5 to the preoperative HGS that rate of HGS became smoother on POD 5. The European System for Cardiac Operative Risk Evaluation (EuroSCORE) is a risk stratification model used to predict mortality after cardiac surgery. We used EuroSCORE II, which considers factors such as age, comorbidities, and surgical urgency to assess the operative risk, in this study.

The primary outcome was whether handgrip recovery (HGS on POD 5/preoperative HGS) was a better predictor of 30-day postoperative complications than was absolute HGS. The secondary outcomes were the lengths of hospital and ICU stays.

The sample size was calculated using a 2-independent-proportion formula according to a previous study.^6^ The authors reported that the estimated complication rate in the normal handgrip recovery group (group 1) was P1 = .05, whereas the estimated complication rate in the low handgrip recovery group (group 2) was P2 = .408. The ratio of participants in the 2 groups (r) was calculated as 0.5 on the basis of the observed proportion (71/141). The alpha error was 0.05, the Z-value was 2.58, and the power (1-b) was 80%, corresponding to a Z-value of 1.28. The calculated sample size was 68 in group 1 and 34 in group 2; after considering a 10% dropout rate, the final sample size was determined as 102 participants. Categorical variables were compared using the chi-square test, whereas continuous variables were compared using mean ± SD or median (interquartile range [IQR]). Multivariate analysis was performed using all independent variables with a *P* value less than .2 in the univariate analysis. Receiver operating characteristic (ROC) curve analysis was performed using postoperative complications as the outcome variable. The area under the ROC curve was calculated to assess the predictive ability of HGS recovery. The optimal cutoff value was determined using the Youden index to maximize the sum of sensitivity and specificity. All analyses were performed using R version 4.2.1.

## Results

**Figure 1:**
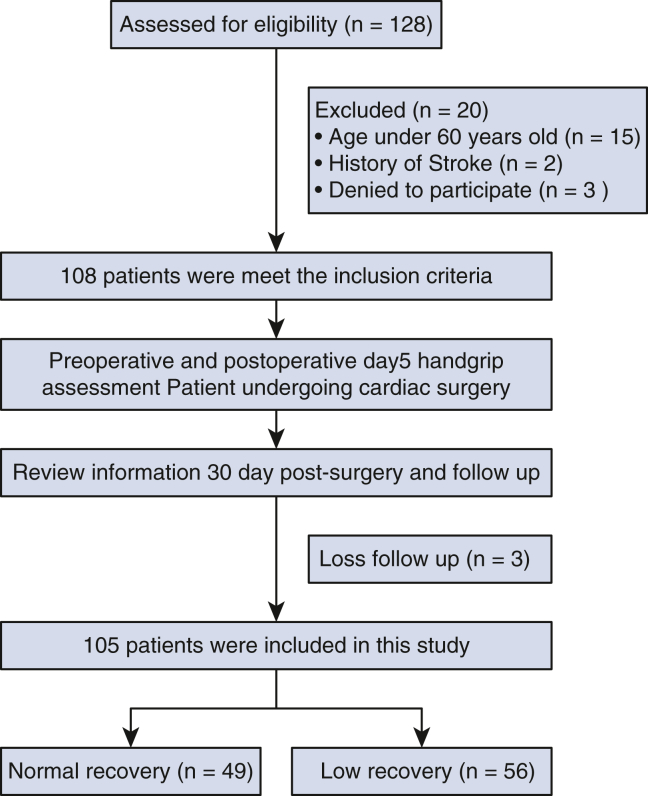
Consort flowchart showing the inclusion of participants in the study.

In total, 128 patients were assessed for eligibility. Twenty patients were excluded for the following reasons: not meeting the inclusion criteria (n = 17), aged less than 60 years (n = 15), history of stroke and having upper arm weakness(n = 2), and refusal to participate (n = 3). Accordingly, 108 patients qualified for the study and completed a preoperative handgrip evaluation before undergoing cardiac surgery. Handgrip assessments were also conducted on POD 5, and follow-up was conducted at 30 days as well. Three patients were lost to follow-up; thus, 105 patients were included in the final analysis (Figure 1).

**Figure 2:**
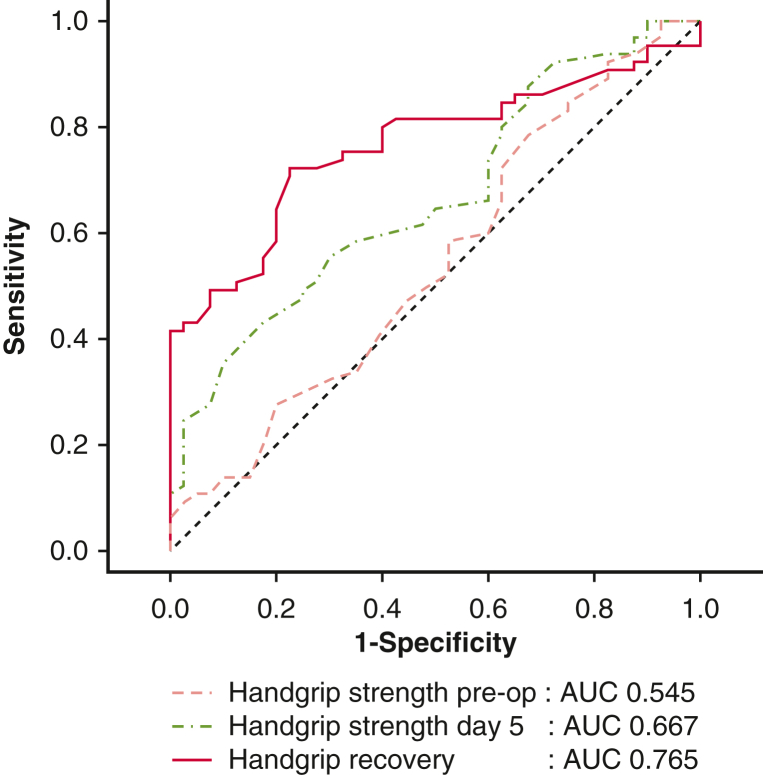
AUCs for preoperative HGS, HGS POD 5, and handgrip recovery. *AUC*, Area under the curve; *HGS*, handgrip strength; *POD*, postoperative day.

ROC curve analysis was performed to assess the predictive accuracy of handgrip recovery, preoperative HGS, and HGS on POD 5 (Table 1). The best predictor of complications was handgrip recovery (area under the curve [AUC], 0.765, 95% CI, 0.92-0.97, *P* < .001; Figure 2). The analysis revealed that the optimal cutoff value for handgrip recovery was 86.67, with a sensitivity of 77.5% and a specificity of 72.3%. In comparison, preoperative HGS was a significantly less valuable predictive variable (AUC, 0.545, *P* = .232). This finding suggested that patients with handgrip recovery of less than 86.67% had a significantly higher risk of complications; thus, this value was a useful clinical threshold. HGS on POD 5 demonstrated a moderate predictive ability, with an AUC of 0.667 (*P* = .002), a sensitivity of 82.5%, and a specificity of 43%.

**Table 1:** Area under the curve and cutoff values to predict the occurrence of complications

Parameter	AUC (95% CI)	*P* value	Cutoff value	Sensitivity (%)	Specificity (%)
Handgrip recovery (%)	0.765 (0.92-0.97)	<.001	86.67	77.5	72.3
Preoperative HGS (kg)	0.545 (0.92-1.02)	.232	30	32.5	78.4
HGS POD 5 (kg)	0.667 (0.88-0.97)	.002	16	82.5	43

*AUC*, Area under the curve; *HGS*, handgrip strength; *POD*, postoperative day.

There were no statistically significant differences among the groups in terms of age, sex, body mass index, smoking, left ventricular ejection fraction, EuroSCORE, medical conditions such as hypertension and diabetes, type of surgery, and the 6-minute walk test. However, dyslipidemia was significantly more prevalent in the normal recovery group than in the low recovery group (79.6% vs 51.8%, *P* = .006). These findings suggested that, other than dyslipidemia, the baseline patients’ characteristics, comorbidities, and surgical procedures were largely comparable between the 2 recovery groups (Table 2). The ICU stay was significantly longer in the low recovery group (median, 4 days; IQR, 2-6 days) than in the normal recovery group (median, 3 days; IQR, 2-4 days; *P* = .007). Likewise, the hospital stay was significantly longer in the low recovery group (median, 8 days; IQR, 7-16 days) than in the normal recovery group (median, 7 days; IQR, 6-8 days; *P* = .002). Complications related to the cardiovascular, respiratory, and neurological systems were significantly more frequent in the low recovery group. Acute kidney injury occurred more frequently in the low recovery group (30.4%) than in the normal recovery group (14.3%); however, the difference was not statistically significant (*P* = .085). Other complications such as infections, reoperations, reintubation, and ICU readmissions were also more frequent in the low recovery group. Specifically, the reoperation and reintubation rates were 17.9% in the low recovery group and 0% in the normal recovery group (*P* = .002). ICU readmission occurred in 10.7% patients in the low recovery group and 0% patients in the normal recovery group (*P* = .029). The overall complication rate was also significantly higher in the low recovery group (83.9%) than in the normal recovery group (36.7%; *P* < .001; Table 3). Logistic regression analysis revealed that low handgrip recovery was strongly associated with a higher likelihood of 30-day complications. As shown in Table 4, the crude odds ratio (OR) was 8.99 (95% CI, 3.59-22.56, *P* < .001). After adjustment for potential confounders, the association remained strong, with a multivariable-adjusted OR of 10.53 (95% CI, 3.92-28.27, *P* < .001) in model 1 and 10.56 (95% CI, 3.09-36.14, *P* < .001) in model 2.

**Table 2:** Characteristics of patients in groups stratified by cutoff values for handgrip recovery

Characteristic	Normal recovery (n = 49)	Low recovery (n = 56)	*P* value
Sex			.374
Male	38 (77.6)	38 (67.9)	
Female	11 (22.4)	18 (32.1)	
Age (y)∗	68 (63,73)	69 (64,72)	.53
BMI (kg/m^2^)†	24 (4.2)	23.6 (3.9)	.661
Smoking	30 (61.3)	27 (48.2)	.333
LVEF (%)∗	58 (43-68)	60 (38-66)	.855
EuroSCORE∗	1.7 (1-2.8)	2.1 (1.2-2.9)	.396
Hypertension	35 (71.4)	40 (71.4)	1
Diabetic mellitus	17 (34.7)	18 (32.1)	.945
Dyslipidemia	39 (79.6)	29 (51.8)	.006
Dominant hand			1
Right	43 (87.8)	50 (89.3)	
Left	6 (12.2)	6 (10.7)	
Type of surgery			.331
CABG	32 (65.3)	30 (53.6)	
Valve surgery	12 (24.5)	14 (25)	
CABG + valve surgery	5 (10.2)	9 (16.1)	
Bentall operation	0 (0)	3 (5.4)	
Duration of surgery (min)∗	355 (315-390)	365 (300-432.5)	.22
CPB time (min)∗	112 (88.8-141.8)	129.5 (97.8-160)	.079
Aortic crossclamp duration (min)∗	74 (56-98)	85.5 (60-120.2)	.086
6MWT (m)†	318.7 (112.2)	276 (106.8)	.152

Data are presented as frequency (percentage) unless indicated otherwise. *BMI*, Body mass index; *LVEF*, left ventricular ejection fraction; *EuroSCORE*, European System for Cardiac Operative Risk Evaluation; *CABG*, coronary artery bypass graft surgery; *CPB*, cardiopulmonary bypass time; *6MWT*, 6-minute walk test.

∗ Data are presented as median (IQR).

† Data are presented as mean ± SD.

**Table 3:** Postoperative complication in the normal and low handgrip recovery groups

Postoperative outcome in 30 d	Normal recovery (n = 49)	Low recovery (n = 56)	*P* value
All complications	18 (36.7)	47 (83.9)	<.001
Cardiovascular system∗	14 (28.6)	31 (55.4)	.01
Respiratory system†	4 (8.2)	16 (28.6)	.016
Neurological system†	7 (14.3)	25 (44.6)	.002
Acute kidney injury	7 (14.3)	17 (30.4)	.085
Infection	0 (0)	4 (7.1)	.121
Reoperation	0 (0)	10 (17.9)	.002
Reintubation	0 (0)	10 (17.9)	.002
Readmission to ICU	0 (0)	6 (10.7)	.029
Readmission within 30 d	1 (2)	7 (12.5)	.065
Length of hospital stay (d)‡	7 (6-8)	8 (7-16)	.002
Length of ICU stay (d)‡	3 (2-4)	4 (2-6)	.007

Data are presented as frequency (percentage) unless indicated otherwise. Neurological complications defined as the need for endotracheal intubation and mechanical ventilation within 72 hours after planned extubation. *ICU*, Intensive care unit.

∗ Cardiovascular complications defined as arrhythmia (including tachyarrhythmia, bradyarrhythmia, or irregular rhythm, occurring postoperatively and requiring medical treatment or intervention) or reoperation.

† Respiratory complications defined as pneumonia (an acute lower respiratory tract infection characterized by new or progressive pulmonary infiltrates on chest imaging, together with clinical features such as fever, leukocytosis or leukopenia, purulent sputum, or worsening oxygenation, requiring antibiotic treatment, or reintubation (defined as the need for endotracheal intubation and mechanical ventilation within 72 hours after planned extubation).

‡ Data are presented as median (IQR).

**Table 4:** Crude and multivariable-adjusted odds ratios for the occurrence of complications at 30 days after surgery, based on the cutoff values for handgrip recovery

Parameter	OR	95% CI	*P* value
Handgrip recovery			
Crude	8.99	(3.59-22.56)	<.001
Multivariable adjusted∗	10.53	(3.92-28.27)	<.001
Multivariable adjusted†	10.56	(3.09-36.14)	<.001

*OR*, Odds ratio.

∗ Adjusted for age, sex, and body mass index.

† Adjusted for age, sex, body mass index, EuroSCORE, duration of surgery, length of ICU stay, and duration of mechanical ventilation, obtained using logistic regression.

Low handgrip recovery was found to be a significant predictor of postoperative cardiovascular, respiratory, and neurological complications within 30 days of surgery. For cardiovascular complications in Table E1 in the Supplement, the crude OR indicated a significant 3-fold increase in risk (OR, 3.1, 95% CI, 1.37-6.99, *P* = .007). This association remained significant after adjusting for age, sex, and body mass index (OR, 3.12, 95% CI, 1.37-7.13, *P* = .006).

In contrast, for the respiratory complications shown in Table E2 in the Supplement, the impact of handgrip recovery remained significant, even after full adjustment. The crude analysis revealed a strong association (OR, 4.5, 95% CI, 1.39-14.58, *P* = .01), which became slightly stronger after adjustment for demographic factors (OR, 4.84, 95% CI, 1.45-16.12, *P* = .005). After incorporation of additional perioperative variables, the association remained statistically significant (OR, 4.44, 95% CI, 1.23-16.06, *P* = .014). This suggested that poor handgrip recovery independently predicted respiratory complications.

Likewise, for the neurological complications listed in Table E3 in the Supplement, low handgrip recovery showed a strong predictive value. The crude analysis revealed an approximately 5-fold increased risk (OR, 4.84, 95% CI, 1.86-12.61, *P* < .001), which further increased after adjustment for demographic factors (OR, 5.81, 95% CI, 2.05-16.48, *P* < .001). Even after full adjustment for perioperative variables, the association remained robust (OR, 4.84, 95% CI, 1.6-14.54, *P* = .003). This indicated that poor handgrip recovery was an independent risk factor for postoperative neurological complications.

## Discussion

In this prospective cohort study, we found that low handgrip recovery was a strong predictor of adverse postoperative outcomes, with the most consistent and independent association with respiratory and neurological complications. Its predictive value for cardiovascular complications was attenuated after adjusting for additional perioperative risk factors. These findings provide new insight into the role of handgrip recovery as a dynamic and clinically relevant assessment parameter and show that it is a superior predictor of postoperative complications than is preoperative or postoperative HGS alone, reinforcing its potential utility in clinical practice.

Low HGS has long been recognized as a surrogate marker of reduced overall muscle strength and physical function, with significant implications for health outcome prediction. Numerous studies have shown that low HGS is associated with an increased risk of all-cause mortality, longer hospital stay, and worse postoperative outcomes. In addition, HGS has been identified as a significant predictor of functional independence in hospitalized patients;^7^ this underscores its clinical relevance for identification of individuals at risk for postoperative complications. Furthermore, research has demonstrated a strong correlation between lower HGS and increased cardiovascular mortality, reinforcing its importance in cardiac surgery.^8^ Vaishya and colleagues^9^ reviewed HGS as a “new vital sign” for health and summarized its association with adverse outcomes such as frailty, mortality, and functional decline in both surgical and nonsurgical populations.

Handgrip recovery is a recovery metric that may provide insights into suboptimal recovery trajectories in patients; therefore, it can guide personalized rehabilitation interventions. Several physiological mechanisms can explain the association between low handgrip recovery and adverse postoperative outcomes. Normal HGS recovery indicates improved muscle reserve and respiratory muscle function, which enhance ventilation and airway clearance, thereby decreasing respiratory complications. However, its association with a reduction in general neurological complications remains limited. Cardiac surgery causes a systemic inflammatory response, with increased levels of proinflammatory cytokines such as tumor necrosis factor-alpha and interleukin-6. These cytokines promote muscle proteolysis and inhibit muscle regeneration.^10^ When combined with the metabolic stress of surgery, this inflammatory response has a more severe impact on frail patients, resulting in prolonged recovery and higher complication rates.

Frailty and sarcopenia, which are well-known geriatric conditions, are significant factors that may compromise postoperative outcomes. The findings of the present study showed that patients with low HGS experienced significantly more complications, in accordance with previous research.^11^ In addition, one study noted that low preoperative HGS was associated with high postoperative complication rates, prolonged hospital stays, and increased mortality risks.^5^^,^^12^ Chung and colleagues^13^ found that low HGS was associated with increased mortality and complication rates after ventricular assist device insertion, consistent with the present findings of prolonged ICU and hospital stays among patients with low recovery. Sarcopenia and frailty are prevalent in elderly individuals and exacerbate the postoperative decrease in muscle strength.^14^ Our cohort was predominantly younger than 70 years, making age-related sarcopenia an unlikely primary explanation. In this population, delayed postoperative HGS recovery more likely reflects reduce physiological and neuromuscular reserve due to acute inflammatory stress, metabolic factors, nutritional status, and subclinical neurological vulnerability. Therefore, HGS recovery serves as an age-independent function marker or resilience to surgical stress and helps identify high-risk patients in the preoperative setting.

Although absolute HGS has been the focus in many studies, the concept of handgrip recovery offers a dynamic perspective on a patient's resilience and capacity for rehabilitation. Our study demonstrated that handgrip recovery (AUC, 0.765) was a stronger predictor of 30-day postoperative complications than was preoperative HGS or postoperative HGS alone. This finding aligns with that of Fu and colleagues,^6^ who revealed a significant relationship between poor perioperative grip strength and 30-day complications after cardiac surgery in middle-aged and older adults. Their study emphasized the importance of monitoring and enhancing grip strength recovery to minimize possible postoperative complications. Although HGS recovery is assessed on POD 5, it reflects the patient's baseline muscle strength and physiological reserve. Patients who fail to recover HGS postoperatively likely had poor preoperative reserve. Therefore, preoperative HGS measurement can be used to identify high-risk patients who are less likely to recover and more likely to develop postoperative complications. Preoperative HGS, although informative, is influenced by age, sex, and body composition, limiting its utility as a stand-alone predictor. Handgrip recovery reflects the physiological and functional changes that occur postoperatively, thus providing a more comprehensive assessment of recovery trajectories.

In this study, the optimal cutoff value for handgrip recovery as a predictor of postoperative complications was determined to be 86.67%, with a sensitivity of 77.5% and a specificity of 72.3%. This cutoff value effectively distinguished high-risk patients who could benefit from targeted interventions. However, the relatively moderate specificity indicates a potential for false positives, necessitating cautious interpretation. Patients with handgrip recovery below this cutoff experienced longer ICU stays, extended hospitalizations, and higher complication rates; these results further support the clinical significance of this threshold. These findings are consistent with those of prior research emphasizing the superiority of dynamic recovery metrics over static strength measurements.

Overall, HGS measurement is simple, inexpensive, and noninvasive, making it an attractive tool for preoperative and postoperative assessments of surgical risk. The findings of this study support the incorporation of handgrip recovery into routine clinical assessments to improve prognosis and enable early therapeutic modulation. Targeted strategies to enhance handgrip recovery can significantly reduce postoperative complications. For instance, prehabilitation programs that focus on resistance training and protein supplementation have been shown to enhance muscle strength and improve surgical outcomes. A prior study showed that patients undergoing prehabilitation had shorter lengths of stay and fewer complications, highlighting the importance of optimizing physical function before surgery.^15^ Postoperative rehabilitation programs should focus on strategies that accelerate muscle recovery. The importance of early postoperative mobilization in terms of shorter hospital stays in elderly patients highlights the need to integrate handgrip recovery metrics into patient care.^16^ Up to 50% of patients experience significant physical inactivity after cardiac surgery. These issues often result in prolonged ICU stays, extended hospitalizations, and increased mortality rates. Early mobilization in the ICU, tailored according to individual patient capacities, can prevent ICU-acquired weakness. Future studies should explore whether targeted interventions in patients with low preoperative HGS can translate these prognostic differences into larger, patient-centered clinical benefits. Engaging patients in preoperative exercise programs can improve functional capacity and expedite postoperative recovery.^17^

### Limitations

Although this study supports the prognostic value of postoperative HGS recovery, several limitations should be acknowledged. First, the observational design reflects routine clinical practice, and patients did not undergo formal prehabilitation programs such as structured exercise or nutritional optimization; therefore, the effect of improving preoperative HGS on postoperative outcomes cannot be determined. Second, frailty was assessed using HGS as a stand-alone measure, without multidimensional frailty tools or comprehensive biological assessments. Although frailty is a multisystem construct, HGS was selected for its clinical practicality as an objective, quantitative, and cost-effective bedside marker; nonetheless, the absence of complementary assessments may limit a more detailed characterization of patient vulnerability. Third, although potential inflammatory mechanisms were discussed, no in-study biomarkers (eg, cytokines or nutritional markers) were collected to validate these pathways, and thus mechanistic interpretations should be considered hypothesis-generating. Fourth, the study was limited by a relatively small number of outcome events. Consequently, some regression estimates demonstrated large ORs with wide CIs, suggesting imprecision and potential sparse-data bias. Fifth, the single-center design and modest sample size may limit generalizability, and larger multicenter studies incorporating biological and frailty markers are warranted. Finally, the relationship between long-term handgrip recovery such as 30-day HGS ratio and postoperative outcomes should be considered in future analyses. Moreover, our follow-up was limited to 30 days postsurgery. Although this period adheres to standard definitions for operative mortality and acute complications, it precludes the assessment of long-term metrics such as 6-month functional recovery or 1-year survival. Future longitudinal studies are required to determine if the prognostic utility of HGS extends beyond the acute perioperative phase.

## Conclusions

Handgrip recovery may be an important predictor of postoperative complications in older adults. Moreover, its measurement is simple and cost-effective, rendering it a reliable parameter for perioperative risk stratification and targeted interventions such as prehabilitation and postoperative rehabilitation. The findings of this study highlight the need to address frailty and muscle recovery for improving surgical outcomes. Further studies with larger cohorts should be conducted, and the mechanisms underlying the predictive value of handgrip recovery should be further investigated.

### Availability of Data and Materials

The datasets used and analyzed during the current study are deidentified and available from the corresponding author on reasonable request.

## Conflict of Interest Statement

The authors reported no conflicts of interest.

The *Journal* policy requires editors and reviewers to disclose conflicts of interest and to decline handling or reviewing manuscripts for which they may have a conflict of interest. The editors and reviewers of this article have no conflicts of interest.

## Figures and Tables

**Table E1:** Crude and multivariable-adjusted odds ratios using the cutoff values of handgrip recovery for cardiovascular complications at 30 days

Parameter	OR	95% CI	*P* value
Handgrip recovery			
Crude	3.1	(1.37-6.99)	.007
Multivariable-adjusted∗	3.12	(1.37-7.13)	.006
Multivariable-adjusted†	1.77	(0.67-4.67)	.245

Obtained by using logistic regression. *OR*, Odds ratio.

∗ Adjusted for age, gender, and body mass index.

† Adjusted for age, gender, body mass index, EuroSCORE, duration of surgery, length of ICU stay, and duration of mechanical ventilation.

**Table E2:** Crude and multivariable-adjusted odds ratios using the cutoff values of handgrip recovery for respiratory complication at 30 days

Parameter	OR	95% CI	*P* value
Handgrip recovery			
Crude	4.5	(1.39-14.58)	.01
Multivariable-adjusted∗	4.84	(1.45-16.12)	.005
Multivariable-adjusted†	4.44	(1.23-16.06)	.014

Obtained by using logistic regression. *OR*, Odds ratio.

∗ Adjusted for age, gender, and body mass index.

† Adjusted for age, gender, body mass index, EuroSCORE, duration of surgery, length of ICU stay, and duration of mechanical ventilation.

**Table E3:** Crude and multivariable-adjusted odds ratios using the cutoff values of handgrip recovery for neurological complications at 30 days

Parameter	OR	95% CI	*P* value
Handgrip recovery			
Crude	4.84	(1.86-12.61)	<.001
Multivariable-adjusted∗	5.81	(2.05-16.48)	<.001
Multivariable-adjusted†	4.84	(1.6-14.54)	.003

Obtained by using logistic regression. *OR*, Odds ratio.

∗ Adjusted for age, gender, and body mass index.

† Adjusted for age, gender, body mass index, EuroSCORE, duration of surgery, length of ICU stay, and duration of mechanical ventilation.
