# Supplementary material for: Association of preoperative handgrip strength and postoperative recovery with outcomes in cardiac surgery patients ≥60 years old
Source: JTCVS Open. 2026 Feb 9;30:101612. doi: 10.1016/j.xjon.2026.101612 (PMC13131115; doi:10.1016/j.xjon.2026.101612)
Supplement: Online Data Supplement [file mmc1.pdf]

Human Research Ethics Committee

Faculty of Medicine, Prince of Songkla University

This document is a record of review and approval/acceptance of clinical study protocol.

**REC.**	66-036-8-1		
**Protocol Title**	Differences in handgrip strength associated with postoperative outcome in older adults undergoing cardiac surgery		
**Principal Investigator**	Jutarat Tanasansuttiporn	**Affiliation**	Department of Anesthesiology, Faculty of Medicine, Prince of Songkla University
**Co-investigator**	Narisara Rachapongthai	**Affiliation**	Department of Anesthesiology, Faculty of Medicine, Prince of Songkla University
	Thadakorn Tantisarasart	**Affiliation**	Department of Anesthesiology, Faculty of Medicine, Prince of Songkla University
	Orarat Karnjanawanichkul	**Affiliation**	Department of Anesthesiology, Faculty of Medicine, Prince of Songkla University
	Suttasinee Petsakul	**Affiliation**	Department of Anesthesiology, Faculty of Medicine, Prince of Songkla University
	Pongsanae Duangpakdee	**Affiliation**	Department of Surgery, Faculty of Medicine, Prince of Songkla University
	Sirichai Cheewatanakornkul	**Affiliation**	Department of Internal Medicine, Faculty of Medicine, Prince of Songkla University
	Warangkana Fongsri	**Affiliation**	Department of Orthopedic Surgery, Faculty of Medicine, Prince of Songkla University
	Khantaros Saelim	**Affiliation**	Department of Anesthesiology, Faculty of Medicine, Prince of Songkla University

Human Research Ethics Committee
Faculty of Medicine, Prince of Songkla University
15 Kamchanavanich Road, Hat Yai, Songkla 90110, Thailand
Tel. 66 7445-1149, 66 7445-1157 Fax: 66 7421-2900

Approved documents:

1. Study protocol version 3.0 date May 25, 2023
2. Participant Information sheet and Informed Consent form version 4.0 date June 8, 2023
3. Clinical report form version 1.0 date January 14, 2023
4. Poster version 2.0 date June 7, 2023
5. Curriculum Vitae and GCP

have/has been reviewed and approved by Human Research Ethics Committee, Faculty of Medicine Prince of Songkla University in full compliance with International Guidelines for human research subject protection such as Declaration of Helsinki, Belmont Report, CIOMS Guideline and the International Conference on Harmonization in Good Clinical Practice (ICH-GCP)

This review is documented in the meeting minutes of the meeting 11/2023, panel 1 agenda 4.2 on April 11 2023

Please submit the Progress Report every 12 months. (Renewal must be submitted at least 30 days prior to expired date.)

(Professor Boonsin Tangtrakulwanich, M.D. PhD.)
Chairman of Human Research Ethics Committee

Date of Approval: June 12, 2023

Date of Expiration: June 11, 2024

Human Research Ethics Committee
Faculty of Medicine, Prince of Songkla University
15 Kamchanavanich Road, Hat Yai, Songkla 90110, Thailand
Tel. 66 7445-1149, 66 7445-1157 Fax: 66 7421-2900
